# Distinct and Conserved Prominin-1/CD133–Positive Retinal Cell Populations Identified across Species

**DOI:** 10.1371/journal.pone.0017590

**Published:** 2011-03-02

**Authors:** József Jászai, Christine A. Fargeas, Sylvi Graupner, Elly M. Tanaka, Michael Brand, Wieland B. Huttner, Denis Corbeil

**Affiliations:** 1 Tissue Engineering Laboratories (BIOTEC), Technische Universität Dresden, Dresden, Germany; 2 DFG Research Center and Cluster of Excellence for Regenerative Therapies Dresden (CRTD), Technische Universität Dresden, Dresden, Germany; 3 Max-Planck-Institute of Molecular Cell Biology and Genetics, Dresden, Germany; Duke University Medical Center, United States of America

## Abstract

Besides being a marker of various somatic stem cells in mammals, prominin-1 (CD133) plays a role in maintaining the photoreceptor integrity since mutations in the *PROM1* gene are linked with retinal degeneration. In spite of that, little information is available regarding its distribution in eyes of non-mammalian vertebrates endowed with high regenerative abilities. To address this subject, prominin-1 cognates were isolated from axolotl, zebrafish and chicken, and their retinal compartmentalization was investigated and compared to that of their mammalian orthologue. Interestingly, *prominin-1* transcripts—except for the axolotl—were not strictly restricted to the outer nuclear layer (i.e., photoreceptor cells), but they also marked distinct subdivisions of the inner nuclear layer (INL). In zebrafish, where the *prominin-1* gene is duplicated (i.e., prominin-1a and prominin-1b), a differential expression was noted for both paralogues within the INL being localized either to its vitreal or scleral subdivision, respectively. Interestingly, expression of prominin-1a within the former domain coincided with Pax-6–positive cells that are known to act as progenitors upon injury-induced retino-neurogenesis. A similar, but minute population of prominin-1–positive cells located at the vitreal side of the INL was also detected in developing and adult mice. In chicken, however, prominin-1–positive cells appeared to be aligned along the scleral side of the INL reminiscent of zebrafish prominin-1b. Taken together our data indicate that in addition to conserved expression of prominin-1 in photoreceptors, significant prominin-1–expressing non-photoreceptor retinal cell populations are present in the vertebrate eye that might represent potential sources of stem/progenitor cells for regenerative therapies.

## Introduction

Vision is one of the most important sensory modality channelling information into the brain through a specialized set of photoreceptors coupled to a chain of second and third order neuronal cells. Although numerous diseases might lead to degenerative alteration of this neuronal circuitry, the most common cause of blindness is the primary degeneration of photoreceptor cells. These pathologies are often of inheritable nature, e.g. retinitis pigmentosa [1] and achromatopsia [2]. Mutations in several genes have been linked to degeneration of photoreceptors (see Retinal Information Network at http://www.sph.uth.tmc.edu/Retnet). One of these, *PROM1* (PROML1; prominin-1) encodes for the prototype of a family of cholesterol-binding pentaspan membrane glycoproteins conserved throughout the animal kingdom [3]–[6].

Previous studies have revealed that prominin-1 (CD133) is highly enriched in precursor structures of mammalian rod photoreceptive disks, i.e. plasma membrane evaginations growing out at the base of the outer segment from the connecting cilium [7]. The importance of prominin-1 for the photoreceptor cell architecture and integrity (particularly that of rods) has been established from four distinct lines of observation. First, recessive and dominant mutations in the *PROM1* gene cause inherited retinal degeneration including retinitis pigmentosa, macular degeneration and cone-rod dystrophy [7]–[11]. Second, the lack of prominin-1 in a murine knockout model leads to complete disorganization of the outer segment early in postnatal development ending with the complete loss of the outer nuclear layer (ONL) in older animals [12]. Third, immunoprecipitation studies have revealed a physical interaction of prominin-1 with protocadherin-21 (PCDH21) [9], whose proteolytic processing of PCDH21 appears essential for the cytoplasmic release of newly synthesized photoreceptive disks [13], [14]. Fourth, a *Drosophila* homolog of prominin-1 has been shown to be involved in defining ommatidial distance of the compound eye through an interaction with spacemaker [15]. Thus, prominin-1 appears to be a key player in maintenance of the integrity of photoreceptors from insects to mammals in spite of the significantly distinct organizing principles of their visual organs [16].

In mammals, besides its above-described role in photoreceptor morphogenesis and maintenance, prominin-1 as a cell surface marker defines a broad range of somatic stem and progenitor cells (reviewed in [6], [17]) including those found in the nervous and hematopoietic system [18]–[20]. It is also associated with embryonic stem cell-derived neural progenitors [21], and importantly, it is detected along the former ventricular zone of the developing midgestation murine retinal primordium containing neuroepithelial progenitors well before retinal histogenesis takes place [7], [18]. However, putative retina–specific prominin-1–positive stem cells seem to be absent from adult individuals since neither primate nor rodent retina have any sign of functional reconstitutive regeneration [22]. In contrast, aquatic anamniote vertebrates (e.g. amphibian and fish) and even embryonic avian species (e.g. chicken) can regenerate and restore visual function efficiently in response to physical, chemical or surgical lesions (reviewed in [23]–[25]). Where this difference originates from, is not yet determined. Nevertheless, this raises an intriguing question whether the retina of highly regenerative non-mammalian vertebrates might harbour a unique cell population with potential capacity to participate in neuronal regeneration. Since there is little information available regarding the anatomical compartmentalization of prominin-1 in eyes of highly regenerative non-mammalian vertebrate species, this prompted us to investigate its retinal distribution in axolotl (*A. mexicanum* [*Am*]), zebrafish (*D. rerio* [*Dr*]) and chicken (*G. gallus* [*Gg*]) by non-radioactive *in situ* hybridization. These vertebrates are often used in experimental modelling of human diseases and regeneration of complex anatomical structures (reviewed in [23]–[26]).

Here we describe that in addition to conserved expression of prominin-1 in photoreceptor cells – depending on the species – additional, non-photoreceptor type prominin-1–expressing cell populations were found being confined to the inner nuclear layer (INL). Surprisingly, a prominin-1–positive cell population associated with INL was also detected in the mammalian (murine) retina while representing only a minute population therein.

## Results

In order to get more insights into the spatiotemporal expression pattern of prominin-1 within the retina of three distinct classes of non-mammalian vertebrate model organisms (e.g. amphibian, fish and avian), we identified cognates of this gene from axolotl, zebrafish and chick and localized their expression using a non-radioactive *in situ* hybridization (ISH) technique, and compared them to the murine eye model.

### Identification and molecular cloning of prominin-1 from non-mammalian vertebrates

By combined molecular and *in silico* cloning approaches we have identified prominin-1 orthologues from axolotl, zebrafish and chicken (for cloning strategy, cDNA sources and molecular details please see Supplemental Materials and Methods S1 and Tables S1, S2 and S3). In contrast to other vertebrates, the zebrafish prominin-1 gene is duplicated and consequently, both co-orthologues of mammalian prominin-1 are referred as to prominin-1a and b [27]. Although their putative ternary structure is well preserved (i.e. pentaspan membrane topology), their overall sequence homology determined by pairwise comparisons with human prominin-1 is rather modest with ≈70 and 45% amino acid similarity and identity, respectively (Table S4).

In agreement with the detection of multiple splice variants of mammalian (rodents and primates) prominin-1 [28]–[31], we have amplified either corresponding or new variants in the three non-mammalian vertebrate models. For instance, in axolotl, in addition to prominin-1 splice variant s1 and s11 (for nomenclature see [30]), three new ones, named s13, s14 and s15, were amplified from a cDNA pool prepared from 3.5–cm long juvenile animal (GenBank entries: DQ285041 to DQ285045; Table S1). They contain an additional insert of four amino acid residues (ALPY) located between exon 10 and 11 according to the mammalian prominin-1 gene structure (Table S1). There is so far no evidence for the presence of this new mini-exon referred to as A10′ in mammalian genes. However, it is alternatively spliced in or out of four *Xenopus tropicalis* prominin-1 expressed sequence tag (EST) sequences (CR573063, CR575948, CN095781 and CX414982) as AIPH sequence, and present in EST sequences of *Oreochromis niloticus* (GR604086) and *Oncorhyncus mykiss* (EZ840481) related to *Dr* prominin-1a, as ALPR and SLPG, respectively. A three amino acid sequence (ALP) is also present in the sequence of *Dr* prominin-1b (GenBank accession number AF373869 and derived NM_198071) and related fish EST sequences from *Gasterosteus aculeatus* either larvae (DT989581) or adult eye (DN678943) and *Oryzias latipes*/medaka (DK100961). In bird, four prominin-1 splice variants were amplified from cDNA templates derived from either embryonic day 5 (E5) chick eye or brain from which two (referred to as s16 and s17) represented novel ones (for details see Table S2; GenBank entries: HQ286789-92). The latter one (i.e. s17) contains the mini-exon A10′ described above in axolotl. In fish, in addition to the prominin-1a splice variant s11, three new variants (designated as s18-20) were amplified from cDNA templates derived from either adult zebrafish retina or brain (for details see Table S3; GenBank entries: HQ386793-96). They contain two facultative exons F7′ and F27′ that were also identified in the distinct *Dr prominin-1b* gene (Table S5). Prominin-1b F7′ exon (VKVESAIK) is spliced into the retina EST sequence BE015742 while F27′ (YPSYDTMRWYPRASAPPRQPDW) is predicted to be located 2573 nt downstream of exon 24 in the genomic sequence and is flanked by canonical donor and acceptor splice sites (data not shown). These results are in line with the high conservation of the genomic organization of *prominin-1* genes among vertebrate species and with other members of the prominin family [4]. *Dr* prominin-1b (AF373869) therefore corresponds to splice variant s21 (Table S5).

Thus, as in rodents and primates, prominin-1 from non-mammalian vertebrate animal models has the same propensity to appear in various alternatively spliced forms being suggestive of multiple — yet unidentified — interacting partners and/or specific functions. It ensues that depending on the species, the stage and the tissue source prominin-1 may display at least ten alternative C-termini (from now on referred as to type A to J) that are summarized in Table 1. Their physiological expression remains to be confirmed using specific antibodies.

**Table 1:** Demonstrated and predicted alternative prominin-1 C-terminal domains‡.

Type	Exons included	C-terminal sequence (species depicted)	Splice variant	Species^reference^
A	24, 26b, 27, 28	(H) RRMDSEDVYDDVETIPMKNMENGNNGYHKDHVYGIHNPVMTSPSQH	s1, s2, *s13*	*A^1^*, H^2,5^, M^3,6^, R^4^
B	24, 26a	(M) RRMDSEDVYDDSSVSGMWHFTL	s3, *s4, s5*	M^7^
C	24	(M) RRMDSEDVYDE	s6	*F^8^*, *H^7^*, M^7^
D	24, 28	(H) *RRMDSEDVYDDPSQH*	*s7, s8, s11, s15, s18, s21*	*A^1^*, *C^1^*, *H^7,9^, F^1,10^*, *M^7^*, *R^9^*
E	24, 25, 26b	(H) RRMDSEDVYDDSSWVTSVQC*	*s9*	*H^9^*
F	24, 26b, 28	(H) *RRMDSEDVYDDVETIPMKNPSQH*	*s10, s12, s14*	*A^1^*, *H^9^*, *Rh^9^*
G	24, 27, 28	(C) RRMDTEDVYDDMENGNNGYHKEHLYGIHNPVITSSVEQW**	*s16, s17*	*C^1^, D^11^, M^12^*
H	24, 26b, F27′	(F) *RRMDTEDVYDDIETFPMKTIPTYDTMTRFPRASAPPRHADW*	*s19, s20*	*F^1,13^*, *A^14^*
I	24, 26b, 27, F27′	(A) *RRMDVYDDVETVPMKNLENGNNGYHNEYLYGIHNPIMTSMSSYDTVNRFPRASAPPRQDD*	ND	*A^15^*
J	24, F27′	(F) *RRMDTEDVYDDIPTYDTMTRFPRASAPPRHADW*	ND	*F^16^*, *A^17^*

‡Alternative splicing might generate ten distinct cytoplasmic C-terminal tails of prominin-1. Italics indicate predicted sequences, in the absence of protein data, for a given species.

Complementary DNA; A, amphibian; C, chicken; D, dog; F, fish; H, human; M, mouse, R, rat, Rh, rhesus.

Asterisk indicates that exon 25 introduces a frameshift on the following exon 26b. Double asterisk indicates that exon 27 in dog harbors a stop codon that would generate a truncated C-terminus in this species (MENGNIGFHRHHSTQTV). ND, not determined.

*References*: 1, present study; 2, Yu et al. 2002; 3, Weigmann et al. 1997; 4, Corbeil et al. 2001; 5, Miraglia et al. 1997; 6, Miraglia et al. 1998; 7, Fargeas et al. 2004; 8, McGrail et al. 2010; 9, Fargeas et al. 2007; 10, Fargeas et al. 2003; 11, *Canis lupus* EST DT541441 and DT541268; 12, *Mus musculus* EST EH097137; 13, *Pimephales promelas* EST DT164241; 14, *Xenopus tropicalis* EST CX838199 and CX912260; 15, *Xenopus leavis* EST BJ061920; 16, *Osmerus mordax* EST EL532935, *Pimephales promelas* EST DT181633, *Cyprinus carpio* EST EX822056, *Salmo salar* EST DW541737; 17, *Xenopus leavis* EST CK796851.

### Cellular distribution of prominin-1 within the non-mammalian eye

#### Axolotl

**Figure 1:**
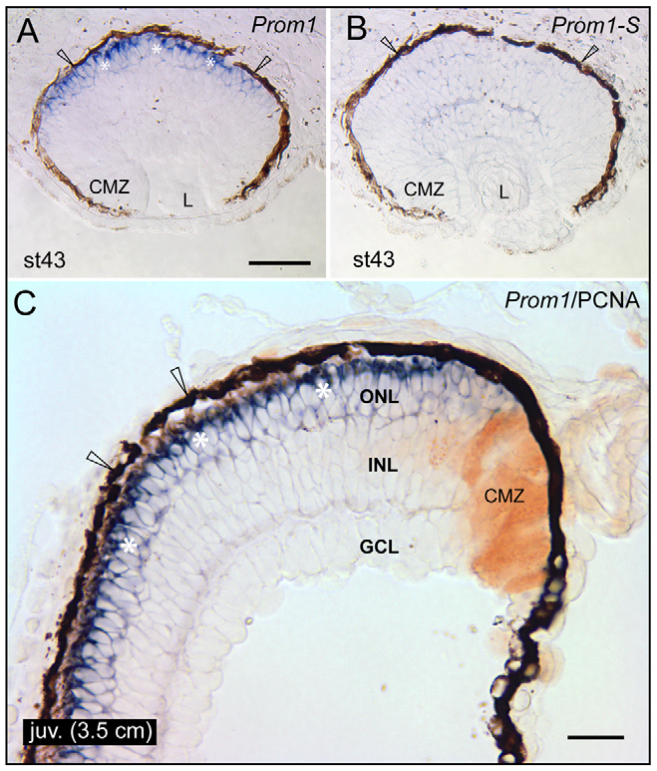
Localization of prominin-1 in the retina of axolotl. (A–C) Cryosections of eyes from larval (A, B; stage 43) and juvenile (C; juv, length of 3.5 cm) axolotls were processed for non-radioactive ISH using either antisense (A, C; *Prom1*) or sense (B; *Prom1-S*) DIG-labelled prominin-1 probe alone (A, B) or combined with IHC detection of proliferating cell nuclear antigen (C; PCNA). Asterisks indicate *prominin-1*–positive cell bodies (A, C, blue) located within the outer nuclear layer (ONL). Arrowheads indicate the retinal pigmented (dark brown) epithelium. Note the lack of *prominin-1* reactivity within the ciliary marginal zone (CMZ), which is labelled with PCNA (C, brown). L, lens; INL, inner nuclear layer; GCL; ganglion cell layer. Scale bars, A and B, 100 µm; C, 50 µm.

The cellular distribution of *prominin-1* transcripts within the eye was investigated in both larval and juvenile axolotl by ISH using a probe that allows the detection of all potential splice variants. In larval stage 43, prominin-1 expression was restricted to the prospective photoreceptor cells occupying the ONL (Fig. 1A, asterisks). In juvenile animals, its detection remained robust and confined to the ONL (Fig. 1C, asterisks). In both larval and juvenile periods, the proliferative ciliary marginal zone (CMZ; Fig. 1A, C), i.e. the peripheral area responsible for the circumferential-appositional growth of the anamniote retina marked by proliferating cell nuclear antigen (PCNA) expression (Fig. 1C, brown), the retinal pigmented epithelium (Fig. 1A, C, arrowheads) and the lens (Fig. 1A, data not shown), were negative. No signal was detected with the sense cRNA probe (Fig. 1B, data not shown).

#### Zebrafish

**Figure 2:**
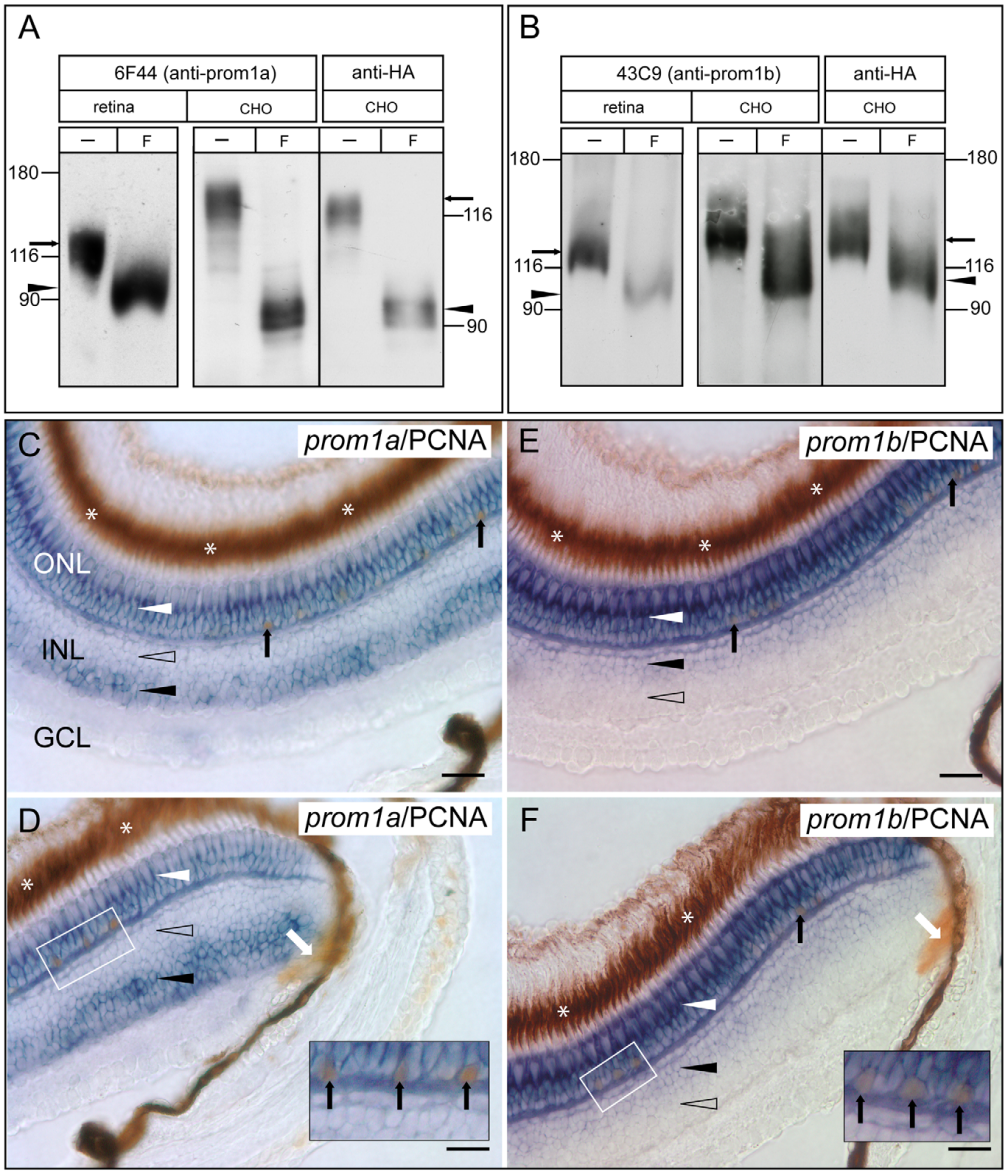
Expression and differential distribution of prominin-1a and b in the retina of zebrafish. (A, B) Lysates prepared from either zebrafish retina (retina) or CHO cells transiently transfected with either *Dr* prominin-1a (A, CHO) or prominin-1b. (B, CHO) both fused in-frame to a C-terminal HA-epitope tag were incubated in absence (–) or presence (F) of PNGase F and analyzed by immunoblotting with either rabbit antiserum 6F44 (6F44; anti-prominin-1a) or 43C9 (43C9; anti-prominin-1b) or rat mAb anti-HA (anti-HA). Arrow and arrowhead indicate native and N-deglycosylated forms of *Dr* prominin-1a (A) or prominin-1b (B), respectively. The position of prestained apparent molecular mass markers (in kDa) is indicated. (C–F) Cryosections of eyes from adult zebrafish were processed for non-radioactive ISH using antisense DIG-labelled prominin-1a (C, D; *prom1a*) or prominin-1b (E, F; *prom1b*) probe combined with IHC detection of proliferating cell nuclear antigen (PCNA). Central (C, E) and peripheral (D, F) regions are presented. White arrowheads indicate *prominin-1a–* and *prominin-1b*–positive cell bodies (blue) located in the outer nuclear layer (ONL). Black and hollow arrowheads indicate the presence or absence of a given *prominin-1* molecule reactivity within the inner nuclear layer (INL), respectively. Black and white arrows point to PCNA–positive nuclei (brown) within the ONL and ciliary marginal zone, respectively. Asterisks indicate the retinal pigmented (dark brown) epithelium. The boxed area demarcates a region displayed at higher magnification in the inset of the corresponding panel, where scarce prominin-1/PCNA–positive cells are observed. Notice that, in addition to the overlapping signals of both zebrafish co-orthologues of mammalian prominin-1 in the ONL, a differential expression could be observed within the INL with respect to the scleral and vitreal subdivisions. GCL, ganglion cell layer. Scale bars, 25 µm.

As described above, the prominin-1 gene in zebrafish is duplicated. Therefore, we have analysed the retinal expression of both zebrafish co-orthologues of mammalian prominin-1 in parallel. First, we assessed their expression by immunoblotting using rabbit antisera raised against a portion of the second extracellular loop of each molecule (for details see Materials and Methods). The immunoblotting of zebrafish retina lysates using either 6F44 (anti-prominin-1a) or 43C9 (anti-prominin-1b) antisera revealed in both cases a broad band with an apparent molecular mass of ≈120 kDa (Fig. 2A, B, retina, arrow). The PNGase F treatment converted them into ≈95 kDa product (Fig. 2A, B retina, arrowhead), which is in agreement with the predicted molecular weight of unglycosylated prominin-1a (90,000) and b (92,500) upon cleavage of signal peptide. The authenticity of the immuno-materials detected was confirmed by the ectopic expression of either recombinant prominin-1a– or b–hemaglutinin (HA) tagged cDNA in Chinese hamster ovary (CHO) cells, and the essentially identical pattern detected with anti-HA antibody (Fig. 2A, B, CHO, respectively). Note that the glycosylation states of recombinant prominin-1 (particularly prominin-1b) are slightly different from that of native proteins being consistent with a tissue-specific glycosylation pattern as reported earlier for the mammalian prominin-1 [29]. No immunoreactivity was observed when CHO cells were transfected with the expression vector alone (data not shown). Thus, both prominin-1 proteins are physiologically expressed in the zebrafish retina.

**Figure 3:**
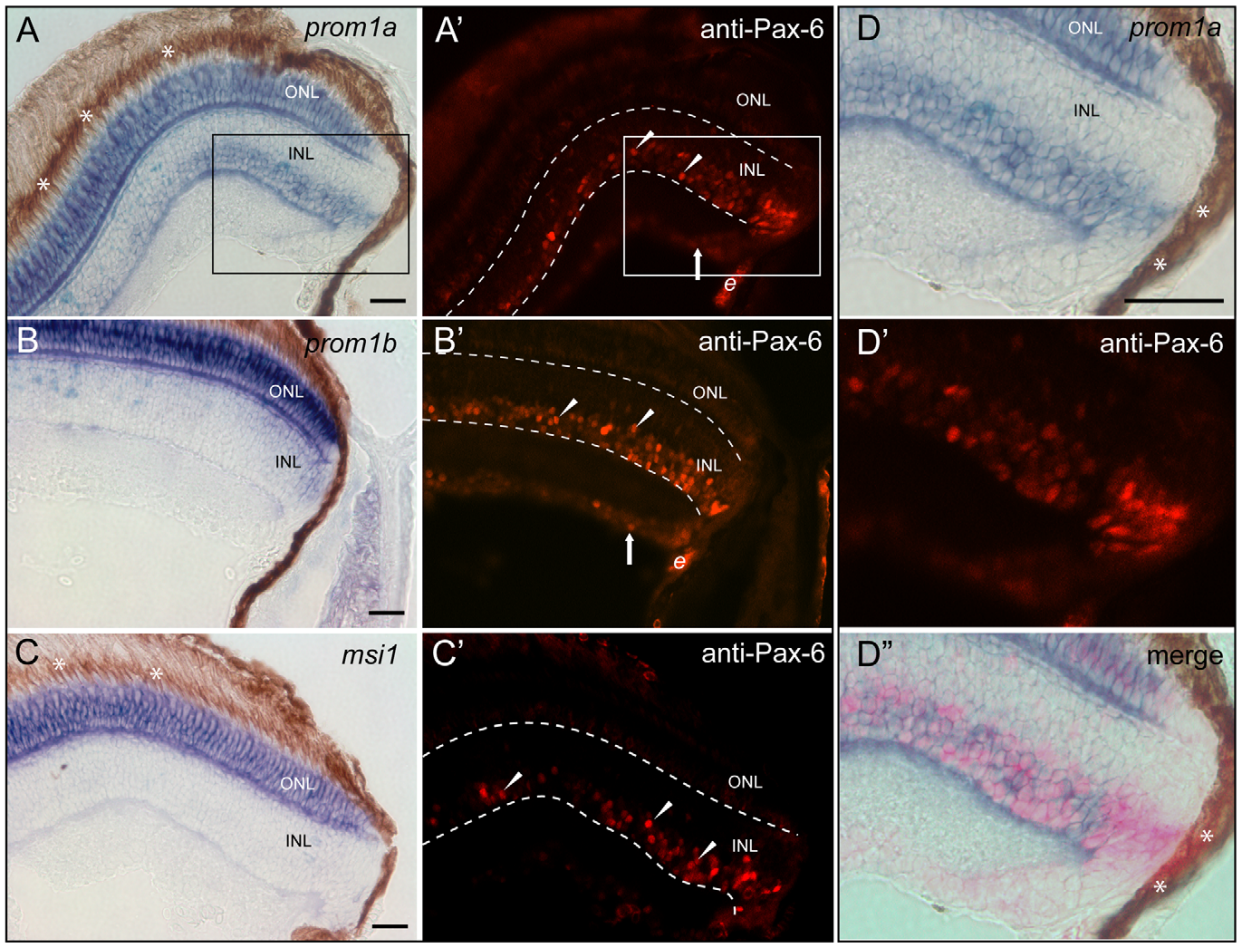
Co-expression analysis of zebrafish prominin-1a or b with Pax-6 and/or musashi-1 within retina cell layers. (A–D″) Cryosections of eyes from adult zebrafish were processed for non-radioactive ISH using antisense DIG-labelled prominin-1a (A, D, D″; *prom1a*), prominin-1b (B; *prom1*b) or musashi-1 (C; *msi1*) probe combined with IHC detection of Pax-6 (A′–C′, D′, D″; anti-Pax-6). Arrowheads and arrows show the Pax-6 immunoreactivity (red) within the inner nuclear layer (INL) and ganglion cell layer (GCL), respectively. The area shown in the inset (A, A′) is displayed as zoom-in in panels D-D″. Note the expression of Pax-6 overlaps with *prominin-1a* within the INL. Asterisks indicate the retinal pigmented (dark brown) epithelium. Dashed lines delineate the INL. e, erythrocytes. Scale bars, 25 µm.

Next, their retinal localization was investigated by ISH using specific probes. Within the ONL of three month-old fish, both prominin-1 molecules were detected (Fig. 2C–F, white arrowhead; Fig. 3A, B) in a similar pattern to that of mushashi-1 (Fig. 3C), a RNA-binding protein required for maintenance of photoreceptor cells [32]. Of the two prominin-1 paralogues, expression of prominin-1b appeared to be more robust in line with gene expression profiling reported earlier on embryonic retina [33]. Interestingly, a scarce sub-population of prominin-1–positive cells located along the vitreal side of the ONL was co-labelled with PCNA indicating prominin-1 expression by proliferating rod precursor cells (Fig. 2C–D, black arrows; see also insets in D, F). Surprisingly, in contrast to axolotl (see above), both prominin-1a and b were detected in the INL with yet a differential expression pattern (Fig. 2A–D, black arrowheads). Prominin-1a was strongly expressed along the vitreal side of the INL (Fig. 2C, D, black arrowheads; Fig. 3A), where amacrine and radial Müller glial cells reside marked by the nuclear expression of Pax-6 (Fig. 3A′, see also inset and panels D-D″), a paired-like homeobox protein [34]. Toward the periphery of the neural retina, the domain of prominin-1a–positive cells abutted the PCNA-labelled proliferating progenitors of the CMZ (Fig. 2D, brown, white arrow). In contrast, prominin-1b was weakly, but reproducibly, detected at the scleral, but not the vitreal, side of the INL (Fig. 2E, F; Fig. 3B), being consequently absent from the Pax-6-labelling domain (Fig. 3B′). Thus, in addition to the photoreceptor cells, two novel sub-populations of prominin-1–expressing cells were detected within the fish retina.

#### Chicken

**Figure 4:**
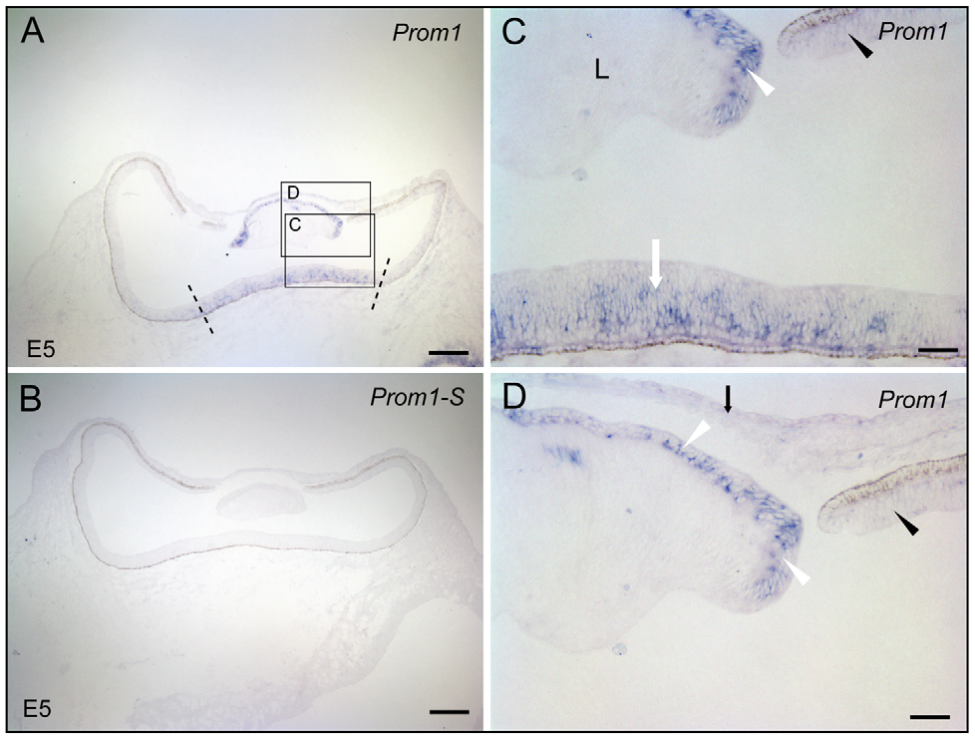
Localization of prominin-1 in the eye of chick at the embryonic day 5. (A–D) Cryosections of chick embryos (E5) were processed for non-radioactive ISH using either antisense (A, C, D; *Prom1*) or sense (B; *Prom-1S*) DIG-labelled prominin-1 probe. The boxed areas in panel A are shown at higher magnification in panels C and D. Dashed lines delimit the central retinal primordium and white arrow indicates prominin-1 expression. Black arrowhead and arrow point to the peripheral retinal primordium and corneal epithelium, respectively, being weakly positive for prominin-1. White arrowheads indicate the *prominin-1*–positive anterior lens (L) epithelium. Scale bars, A and B, 250 µm; C and D, 50 µm.

**Figure 5:**
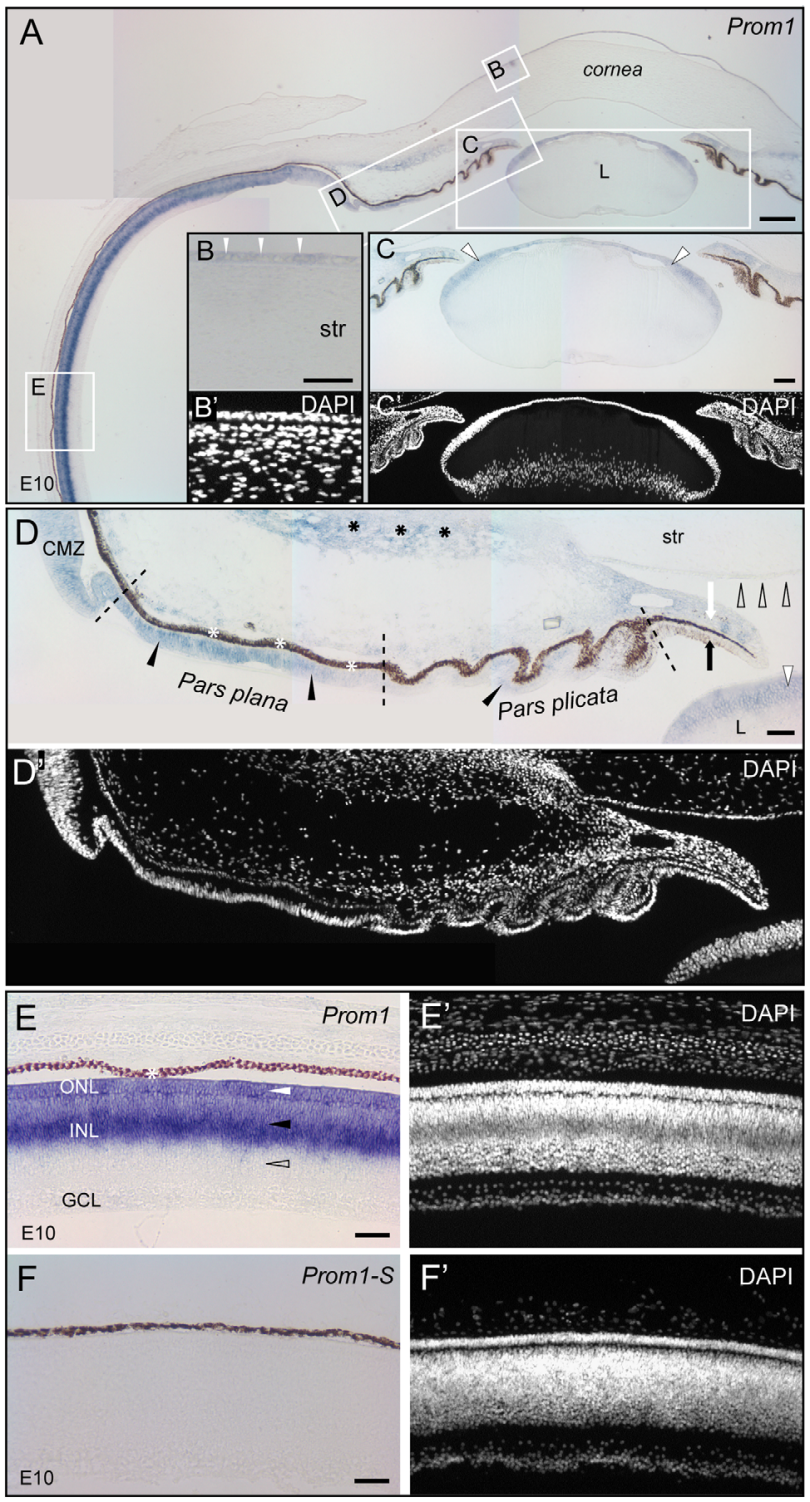
Localization of prominin-1 in the eye of chick at the embryonic day 10. (A–F′) Cryosections of eyes from chick (E10) were processed for non-radioactive ISH using either antisense (A–E; *Prom1*) or sense (F; *Prom1-S*) DIG-labelled prominin-1 probe. Sections were counterstained with DAPI (B′–F′). (A) An overview of the anterior- and parts of the posterior eye segment is shown. The boxed areas in A are shown at higher magnification in panels B–E. (B) White arrowheads indicate the weak expression of prominin-1 in the corneal epithelium. Note the lack of signal in stromal cells (str). (C, D) Framed white arrowheads indicate prominin-1–expressing anterior epithelial cells of the lens (L). Dashed lines (D) indicate the putative border between distinct compartments of the prospective blind part of the retina. The anterior (*pars plicata*) and posterior (*pars plana*) regions of the non-pigmented layer of the ciliary epithelium are weakly and strongly positive for *prominin-1* transcripts (black arrowheads), respectively. The ciliary marginal zone (CMZ) is positive for prominin-1. Black hollow arrowheads indicate weakly expressing prominin-1–positive cells in the corneal endothelium. White arrow points to the stroma of the iris harboring *prominin-1* transcripts whereas black arrow indicates the prospective inner layer of the iris epithelium being negative. Black asterisks indicate *prominin-1* transcripts in mesenchymal cells giving rise to the ciliary body. (E) White and black arrowheads indicate the prominin-1 expression in the outer nuclear layer (ONL) and scleral side of the inner nuclear layer (INL) of the retina, respectively. Hollow arrowhead indicates the lack of prominin-1 signal in the vitreal side of INL. White asterisk, retinal pigmented (dark brown) epithelium; GCL, ganglion cell layer. Scale bars, A, 250 µm; B, C, 100 µm; D, 50 µm; E, F, 25 µm.

Series of developmental stages of chicken - ranging from E5 to E20 - were analyzed for prominin-1 expression. At the earliest stage analyzed (i.e. E5), *prominin-1* transcripts were enriched in neuroblasts of the central part of the retinal primordium (Fig. 4A, C, white arrow). A faint staining was also seen toward the periphery of the primordium that gives rise during development to the inner ciliary/iris epithelium (Fig. 4A, C, D, black arrowhead). Prominin-1 expression was however not restricted to the neuroectoderm-derived retinal primordium. An accumulation of transcripts was noted in the subcapsular anterior epithelium of the developing lens (Fig. 4A, C, D, white arrowhead). By E10, the development of the anterior ocular structures is significantly advanced and they are well discernable (Fig. 5A). Therein, the expression of prominin-1 highlights the corneal epithelium (Fig. 5B, white arrowheads), the subcapsular anterior lens epithelium (Fig. 5C, white arrowheads), the mesenchyme of the developing *iris* (Fig. 5D, white arrow) and weakly the corneal endothel (Fig. 5D, black hollow arrowheads). In the retina, which displays at that time the three cellular layers, prominin-1 expression was more widespread covering also its peripheral parts encompassing the CMZ as well as the prospective non-pigmented inner ciliary epithelium (Fig. 5D, black arrowheads). Within the differentiating sensory/neural retina, a robust expression was detected within two domains. Just like in amphibian and fish, one of those compartments was the ONL (Fig. 5E, upper white arrowhead). The second one was the INL where prominin-1–expressing cells were aligned along the scleral side leaving approximately the vitreal one-third of the layer negative (Fig. 5E, E′, black and hollow arrowhead, respectively). The latter staining is reminiscent of the distribution *Dr* prominin-1b in the INL (see above).

At later stages of incubation (e.g. E15 and E20), retinal distribution of prominin-1 was similar to that of stage E10 (Figs. S1 and S2). However, the intensity of the staining was overall weaker. The vitrealmost side of the INL remained negative for prominin-1 expression (Figs. S1 and S2, hollow arrow).

### Cellular distribution of prominin-1 within the mammalian eye

To date, the expression of prominin-1 protein in the murine retina was reported to be confined to photoreceptor cells [7], [12]. In light of the data described above, we re-analysed its cellular localization in fetal, post-natal and adult mice by ISH to set the stage for further comparative studies concerning its compartmentalization across the vertebrate *Phylum*.

**Figure 6:**
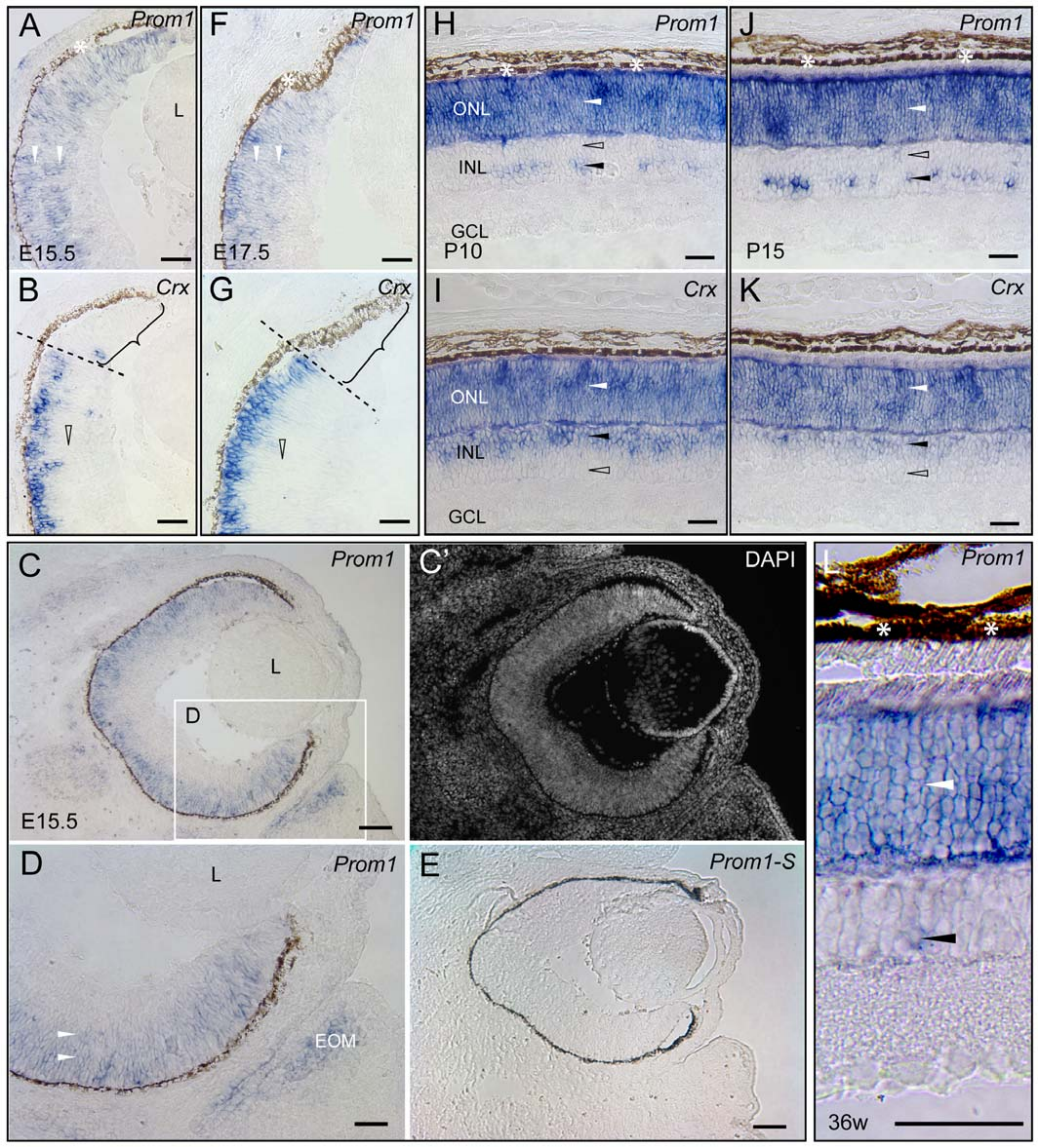
Localization of prominin-1 in the eye of mice. Cryosections of eyes from embryonic (E15.5, E17.5), postnatal (P10, P15) and adult (36 weeks) mice were processed for non-radioactive ISH using either antisense (A, C, D, H, J, L; *Prom1*) or sense DIG-labelled prominin-1 (E; *Prom1–S*) or Crx (B, G, I, K; *Crx*) probe. Section was counterstained with DAPI (C′). The boxed area in panel C is displayed at higher magnification in panel D. (A–G) White arrowheads indicate the broad prominin-1 expression within the retinal primordium at early developmental stages whereas the hollow arrowhead and bracket indicate the lack of Crx expression in the neuroblasts and the prospective ciliary/iris epithelium, respectively. Dashed lines indicate putative border between the prospective optic and blind part of the retina. Beside the retinal primordium, prominin-1–expressing cells are found in the extraocular mesenchyme (EOM). The developing lens (L) devoid of prominin-1. (H, K) White and black arrowheads indicate the expression of prominin-1 or Crx in the outer nuclear layer (ONL) and the inner nuclear layer (INL) of postnatal animals, respectively. Hollow arrowheads indicate the lack of prominin-1 or Crx in a particular side (scleral or vitreal) of INL. Note the scarce prominin-1–expressing cells located along the vitreal side of the INL and the absence of Crx therein. (I) White arrowhead indicates the strong prominin-1 expression in the ONL and a black one points to the rare prominin-1–positive cell found in the vitreal side of the INL of an adult retina. White asterisk, retinal pigmented (dark brown) epithelium. Scale bars, A, B, D, H–L; 50 µm; C, C′, E; 250 µm.

During fetal development (e.g. E15.5 and E17.5), *prominin-1* transcripts were broadly spread within the growing retina. They were found in both neuroblasts and young morphologically undifferentiated photoreceptor cells accumulated along the former ventricular zone of the optic cup (Fig. 6A, F, white arrowheads). The latter zone was marked by the expression of the cone rod homeobox protein *Crx* (Fig. 6B, G), a marker known to regulate the development of photoreceptor cells [35]. Prominin-1 was also detected in the prospective inner ciliary/iris epithelium *(pars caeca retinae)* (Fig. 6A, F) that lacked *Crx* (Fig. 6B, G, bracket). In addition, *prominin-1* transcripts were detected in the extraocular mesenchyme (Fig. 6C, D, EOM). The retinal pigmented epithelium and the developing lens were negative (Fig. 6A, C and D).

In postnatal stages (e.g. P10 and P15), prominin-1 was detected in two of the three cellular layers of the sensory/neural retina. Consistent with its early immuno-detection in photoreceptor cells [7] *prominin-1* transcripts were detected in the ONL (Fig. 6H, J, white arrowhead) where photoreceptor cells being marked by *Crx* expression (Fig. 6I, K, white arrowhead). Interestingly, a second population of prominin-1–expressing cells was detected at the vitreal side of the INL (Fig. 6H, J, black arrowhead). These prominin-positive cells neither formed a contiguous cell layer nor intermingled with displaced photoreceptor cells that are positioned along the scleral side of the INL and marked by the expression of *Crx* (Fig. 6I, K, black arrowhead).

Finally, in adult mice (e.g. 36 week-old) the *prominin-1* transcripts were observed, as in earlier stages, in the ONL and surprisingly, also in rare cells found the INL (Fig. 6L, white and black arrowheads, respectively). Collectively, these data demonstrate that the expression of prominin-1 is not solely restricted to photoreceptor cells of the eye.

## Discussion

The present study reports three major observations concerning the expression and anatomical compartmentalization of prominin-1 in the retina across distinct vertebrate model organisms. First, it is expressed in an evolutionarily conserved fashion in photoreceptors. Second, novel prominin-1–expressing cell populations were detected within the INL in all examined animals with the notable exception of the axolotl. Third, more than one prominin-1 splice variants are expressed in eyes as shown here for the fish and chicken.

### Prominin-1, a common denominator of photoreceptor cells

The retina of vertebrates is remarkably conserved during evolution, both in terms of major cell classes and laminar anatomical organization. The specification and differentiation of retinal cells are orchestrated by an evolutionarily conserved genetic network, which is influenced by both cell-autonomous [36] and non-autonomous [37] cues. During development, six distinct neuronal and one glial cell-types arise from common progenitors in ordered successive, yet overlapping, waves [38]. Certain aspects differ between retinas of distinct vertebrate species, like for instance, the proportion of photoreceptor subtypes (e.g. cone versus rod) that are responsible for photopic or scotopic vision or their architectural details (e.g. opened versus closed disks) varies considerably among taxa [39]–[42]. Nevertheless there are significant functional and morphological similarities, and presence of prominin-1 appears to be a common denominator of photoreceptor membranes throughout the animal kingdom.

Structurally, photoreceptors are forming a complex system of membrane specializations coupled to a dynamic turnover of the outer segment membrane [43]. In these cells, prominin-1 might be somehow involved in the orientation and/or alignment of two adjacent newly synthesized disk precursors at the base of the rod outer segment, or in the general alignment of all open photoreceptor membranes in the cone (see also Introduction) [7], [9], [12], [16]. The physical interaction of mammalian prominin-1 with protocadherin-21, a photoreceptor-specific adhesion molecule, appears indispensable for the organization of the rod outer segment [9]. Irrespective of the animal organism investigated prominin-1 seems to be a key player in the maintenance of the integrity of photoreceptor membranes. Given the increasing number of retinal diseases associated with mutations in *PROM1* gene, it is important to identify all the putative prominin-1 interacting partners (lipids or proteins) [9], [44], [45], and cone-dominant zebrafish retina should be well suited to reveal those implicated in cone morphogenesis. Likewise, it will be of interest to determine whether a genetic interaction of RNA-binding protein musashi-1 with prominin-1 occurs given that both of them are distributed in a similar manner within the ONL. Like prominin-1, musashi-1 appears indispensable for the maintenance of photoreceptors, and mice lacking it suffer from retinal degeneration mimicking retinitis pigmentosa [32].

### Distinct cell subpopulations expressing prominin-1 within retina

We have observed here that expression of prominin-1 cognates was not strictly restricted to photoreceptors as these transcripts were detected in other retinal cell subpopulations located in the INL of different species (e.g. in zebrafish, chick and mouse) with the notable exception of axolotl (see below). This complex compartment harbors several distinct neuronal cell types as well as Müller glial cells [46]. The lack of detection of prominin-1 protein within the INL in early studies [7], [12] might be explained by the fact that its expression domain is far away from perikarya of INL-associated cells. The selective subcellular localization of prominin-1 in various types of plasma membrane protrusion (e.g. microvillus, cilium, filopodium) [3], [18], [20], [29] might be the underlying reason of such phenomenon as demonstrated in mature photoreceptors. As mentioned above, prominin-1 is concentrated at the plasma membrane evaginations growing from the connecting cilium at the base of the outer segment [7], i.e. outside of the ONL where its transcript is detected. Thus, the differential localization (transcript versus protein) is probably one of the main reasons why non-photoreceptor prominin-1–expressing cells were not characterized until now. Moreover, it cannot be excluded that prominin-1 epitope might be somehow masked in INL-associated cells, and drastic techniques for antigen retrieval should be applied.

Thus, INL surprisingly represents a facultative prominin-1–expressing domain with class and species specific features. For instance, both *Dr* prominin-1b and *Gg* prominin-1 were found at the scleral side of INL that contains horizontal and bipolar neuronal cells, whereas *Dr* prominin-1a and *Mm* prominin-1 were restricted to its vitreal side that contains amacrine and Müller glial cells. Interestingly, the latter domain of expression coincides with Pax-6, a master regulator of eye/retinal development [34], [47]. Pax-6 is involved among others in maintaining proliferating immature retinal progenitor populations [47], and its absence results in the small-eye (Sey) phenotype in mice [48]. In the context of growth-associated neurogenesis and neural regeneration, expression of Pax-6 defines a pool of Müller glial cells with dual functions; i) as neurogenic radial glia generates for instance late stage rod precursors that are dispersed within the ONL of adult teleost retina [25], [49], and ii) as latent source of multipotent stem cells that are stimulated during injury-induced replenishing of lost cells in the vertebrate retina [50], [51]. Although the expression of *Dr* prominin-1a has not been evaluated upon injury, the scarce sub-population of prominin-1–positive cells co-labelled with PCNA within the ONL is consistent with its expression in actively dividing rod precursors being descendants of slowly proliferating Müller cells [51], [52]. Moreover, knowing that a subpopulation of murine glial fibrillary acidic protein (GFAP)–positive astrocytes co-express prominin-1.s1 variant [53] and certain astrocytes might function as stem cells in adult mammalian brain [54], the present data suggest that a fraction of INL-associated glial cells highlighted for instance by prominin-1 might act as progenitors within the neural retina. As a marker of stem/progenitor cells being illustrated here by its early detection in chick and murine neuroblasts (see Figs. 5D, 6A, respectively), prominin-1 protein might be utilised to isolate prospectively, and hence, enrich for putative photoreceptor precursors. This is an exciting avenue since transplantable photoreceptor progenitors can integrate into the adult or degenerating mouse retina when taken from the developing retina at the time of ongoing rod genesis [55], [56]. In this perspective, a recent report has shown that rare prominin-1–positive cells within adult human postmortem retina exhibited a neurosphere-forming potential indicating that even in primate retinae exist cells that could eventually serve as cellular source for replacement therapy [57]. In adult mammals, the progenitor properties of these potential somatic retinal progenitor cells might be blocked by environmental cues. A further characterization of different prominin-1–positive cell subpopulations across species in terms of co-expression with lineage-specific markers and their progenitor properties is of utmost importance. In this respect, the present study provides not only a comparative framework but also a tool-box to identify essential factors regulating the biogenesis of the retina and eventually its regeneration.

Although prominin-1 is largely considered as a general marker of stem and progenitor cells, our current analysis indicates that the multipotent progenitors found at the peripheral part (i.e. CMZ) of the teleost fish and amphibians retinae being responsible of growth-associated retino-neurogenesis in adults [25], [58], [59], did not express it. In zebrafish, prominin-1 expression might be turned on upon the exit from CMZ, and thus expressed in slowly proliferating cells within the INL. Lineage-tracing experiments might be useful to solve this issue. In axolotl, the absence of prominin-1–positive cells within both CMZ and INL could reflect that in amphibians the retinal pigmented epithelium appears to play a pivotal role as the cellular source for regeneration through a process of cell de-differentiation [60], [61]. It will be interesting to evaluate whether the expression of prominin-1 might be regulated upon injury, in a way similar to what has been observed during epimorphic regeneration of the spinal neural tube (J.J. and D.C., unpublished data).

Beyond the neural retina, numerous other cells within the developing eye appear to express prominin-1 (see also [27]), which is consistent with its detection in various growing epithelia of early mouse and human embryos as well as in fully differentiated adult epithelial and non-epithelial cells [7], [18], [29], [62]–[67]. The absence or presence of prominin-1 in a given tissue in distinct species needs to be scored with caution as the lack of prominin-1 might coincide with the expression of its paralogue – prominin-2 [4]. Thus, a complete catalogue describing the expression of all members of the prominin-family among species of interests is needed.

### Prominin-1, gene duplication and splice variants

As recently reported by McGrail et al. (2010) *prominin-1* gene duplication in zebrafish likely arose with the whole-genome duplication within the teleost lineage resulting in multiple copies of many genes [68], [69]. Interestingly, the expression of the *Dr* co-orthologues of mammalian prominin-1 at unique sites within the INL suggests a lineage specific sub-functionalization, beside the potential functional redundancy at the overlapping domains within the photoreceptor cells. In addition, our analysis reveals the existence of alternatively spliced prominin-1 variants in non-mammalian vertebrates. At least in two species (i.e. fish and chick) the eye expresses more than one splice variants (see Supplemental Materials and Methods S1), which is in line with previous observations documenting that both rodent and primate might generate numerous prominin-1 splice variants [29]. Indeed, twelve prominin-1 splice variants have been detected and characterized in mammals [30], [31], yet the list is most likely not comprehensive. The functional significance of this variability is not yet understood, but the appearance of certain variants appears to be ontogenetically regulated [53] and/or tissue specific [29], [31]. Recently, several splice variants have been detected in the human retina, prominin-1.s11 being highly expressed therein [11]. This variant that is characterized by a rather short cytoplasmic C-terminus (Table 1; type D, exon 24 and 28) and the presence of a nine amino acid insert (exon 3) within the first extracellular domain (Table S5) [30] is closely related those detectable in the zebrafish and chicken eye. Their precise subcellular localization will require specific antibodies. It is nevertheless interesting to find that such splice variant exists not only in the eye of tetrapods.

### Conclusion

In conclusion, we have demonstrated the conserved expression of prominin-1 in photoreceptor cells among vertebrates, and highlighted novel prominin-1–positive cell populations within the INL that might act under certain circumstances as progenitor cells. Further studies are necessary to fully characterize at the cellular level their potential role in tissue integrity and regeneration, and the utility of prominin-1 as a cell surface marker for isolating these cells.

## Materials and Methods

### Ethics Statement

All animal procedures were performed in compliance with the UK Animals Act (Scientific Procedures) 1986. All efforts were made to minimize the suffering of animals. Animals for organ removal were killed according to S1 method carried out at the Max-Planck-Institute of Molecular Cell Biology and Genetics (Dresden), which holds a license for keeping and breeding of laboratory animals (Erteilung und Erlaubnis für das Züchten und Halten von Wirbeltieren zu wissenschaftlichen Zwecken), issued on 30.1.2001 by Regierungspräsidium Dresden (Az.: 74-9165.40-9-2000-1). The Institute holds a license (Anzeigepflichtige Tierversuche) for collecting organs and tissues (Az.: 74-9168.25-9-2001-1, issued 23.7.2001 by Regierungspräsidium Dresden).

### Molecular cloning of prominin-1 from non-mammalian vertebrates

Nucleotide and protein databases were searched at the National Center for Biotechnology Information using BLAST network services. ScanProsite searches were performed at the ExPaSy Molecular Biology www server [70]. Prominin-1 cDNA sequence was cloned from axolotl by means of degenerated polymerase chain reaction (PCR) and rapid amplification of cDNA ends (RACE). Similarly, a chicken orthologue was identified by a combination of *in silico* methods and degenerated PCR. A partial sequence encoding for *Dr* prominin-1a (formerly prominin-like 1; GenBank Accession Number AF160970) was identified by expressed sequence tag (EST) database search [4]. Its missing 3′-end was amplified by RACE. Finally, the full-length coding sequence of prominin-1 molecules including novel splice variants were amplified using cDNA templates prepared from the following tissues: whole 3.5–cm long juvenile axolotl, E5 chick eye or brain and adult zebrafish retina or brain. A complete coding sequence for *Dr* prominin-1b (formerly prominin-like 2; AF373869) was identified by EST database search [4]. The details of the molecular cloning, cDNA sources and the analysis of various prominin-1 splice variants can be consulted among Supplemental Materials and Methods S1 and Tables S1, S2, S3, S4, and S5 accessible through the website of the journal.

### Plasmid construction

Bacterial expression plasmids pGEX-7M-zfprom-1a and pGEX-7M-zfprom-1b containing a cDNA fragment of either *Dr* prominin-1a (nucleotide (nt) 2111 to 2308 corresponding to residues Gly_631_-Gln_696_; HQ386793) or prominin-1b (nt 2167 to 2364, residues Gly_650_-Gln_706_; AF373869), respectively, each fused in-frame to glutathione S-transferase (GST) cDNA, were constructed by selective PCR amplification of the corresponding cDNA (see above). For pGEX-7M-zfprom-1a, the oligonucleotides 5′-GTTCCGCGTGGATCCGGCATTAATGAGATCGACTTTGCTG-3′ [F-jj-751] and 5′-GACTGCACTCGAG *TCA*CTGCTCCAGTGGGACGAC-3′ [R-jj-752] were used as 5′ and 3′ primers, respectively; for pGEX-7M-zfprom-1b, the oligonucleotides 5′-GTTCCGCGTGGATCCGGCATTGGAGACATTGACTACCAG-3′ [F-jj-753] and 5′- GACTGCACTCGAG *TCA*CTGTTCCATTGGAACCACTTGATC-3′ [R-jj-754] were used. The 5′ and 3′ primers created a *Bam*HI or *Xho*I restriction site (sequence underlined), respectively. In addition, the 3′ primer introduced a TGA stop codon (italic). The amplified fragments were digested with *Bam*HI and *Xho*I and cloned into the corresponding sites of pGEX-7M vector (Pharmacia).

Eukaryotic expression plasmids pEGFP-N1-zfprom-1a-HA and pEGFP-N1-zfprom-1b-HA containing full-length coding sequence of either *Dr* prominin-1a.s11 (HQ386793) or prominin-1b.s21 (AF373869), respectively, were constructed by selective PCR amplification of the corresponding cDNA (see above) each fused in-frame to a C-terminal HA-epitope tag nucleotide sequence. For pEGFP-N1-zfprom-1a-HA, the oligonucleotides 5′-GTCAGATCCGCTAGCTAATCATGCTTTGGAAAACGGCACTG-3′ [F-jj-711] and 5′-TGCAGGATCC *TCA* **GGCGTAGTCGGGGACGTCGTAGGGGTA**ATTCCAGTGTCCAATATCGTCGTC-3′ [R-jj-718] were used as 5′ and 3′ primers, respectively; for pEGFP-N1-zfprom-1b-HA, the oligonucleotides 5′-GTCAGATCCGCTAGCAACATGTGGTGGAAAGTGGGAC-3′ [F-jj-704] and 5′-GACTGCACTCGAG *TCA* **GGCGTAGTCGGGGACGTCGTAGGGGTA**GCTCCAGTTCTCAGAACCATCATC-3′ [R-jj-652] were used. Both 5′ primers contained an *Nhe*I restriction site (sequence underlined) whereas the 3′ primers carried either a *Bam*HI (in case of prominin-1a) or an *Xho*I recognition site (in case of prominin-1b) allowing for directional sub-cloning into pEGFP-N1 vector (Clontech) digested with the corresponding restriction sites. In addition, 3′- primers introduced the coding sequence of HA tag (27 nucleotides double underlined) and a TGA stop codon (italics). In all cases, the amplified PCR DNA fragments were sequenced in order to confirm the absence of any reading mistakes introduced by the DNA polymerase.

### Antisera against recombinant prominin-1

*Escherichia coli* (BL21 strain) was transformed with either plasmids pGEX-7M-zfprom-1a or pGEX-7M-zfprom-1b. Fusion proteins were purified on glutathione Sepharose 4B beads (Pharmacia) according to the batch method as described in the manufacturer's protocol. The fusion proteins were eluted from glutathione Sepharose 4B beads at room temperature by addition of 10 mM glutathione in 50 mM Tris-HCl, pH 8.0. The eluted proteins were mixed (1∶1) with complete Freund's adjuvant and then used to generate antisera 6F44 and 43C9 against *Dr* prominin-1a and prominin-1b, respectively, by immunizing New Zealand white rabbits.

### Tissue samples

#### Mouse

Murine tissue samples were obtained from NMRI strain. Mice were deeply anesthetized by a single intra-peritoneal bolus injection of Ketamine and Xylazine mixture. Animals were then trans-cardially perfused with ice-cold 4% paraformaldehyde (PFA). Eyes were removed and post-fixed in 4% PFA for 2 h at 4°C. After cryoprotection with 30% sucrose-PBS tissue samples were embedded in OCT compound (Tissue Tek, Sakura, The Netherlands). Samples were sectioned on a cryostat (HM560, Microm International GmbH, Walldorf, Germany) at 10 µm and then mounted onto SuperFrost® Plus microscope slides (Menzel-Gläser, Braunschweig, Germany), dried overnight at room temperature, and stored at −20°C until use.

#### Chicken

Fertilized White Leghorn eggs were purchased from an aviary (Cuxhaven, Germany). Eggs were incubated in a humidified egg chamber at 38°C until E5 (HH27 stage according to Hamburger and Hamilton [71]), E10 (HH36), E15 (HH41) and E20 (HH45). On the indicated day of incubation whole embryos were harvested. E5 whole embryos and heads of E10 embryos were fixed by immersion in 4% PFA overnight at 4°C. Embryos of more advanced stages (i.e. E15 and E20) were trans-cardially perfused with ice cold 4% PFA. Eyes were dissected from the orbit and post-fixed in 4% PFA for 2 h at 4°C. After cryoprotection with 30% sucrose-PBS, tissue samples were embedded in OCT compound (Tissue Tek), sectioned at 14 µm and then mounted as above.

#### Axolotl

Neotenic salamanders were bred in the facility of MPI-CBG, Dresden. The animals were kept at 18°C in tap water and fed daily with *Artemia nauplia*. Eye samples were obtained by dissection from larval and juvenile axolotl following an overdose of ethyl-p-aminobenzoate (E-1501; Sigma) dissolved in water at 0.03%. Eyes were fixed by immersion in 4% PFA overnight at 4°C. After cryoprotection with 30% sucrose-PBS, tissue samples were embedded in OCT compound (Tissue Tek). Samples were sectioned at 14 µm and then mounted as above.

#### Zebrafish

Fish were raised and kept under standard conditions at 27°C [72], [73] in the fish facility of the MPI-CBG, Dresden. Adult, sexually matured 3 months old fish from the AB genetic background were used [74]. Eyes were removed from the *orbita* with fine-tip watchmakers forcipes following an overdose of MS222. Tissue samples were fixed by immersion in 4% PFA overnight at 4°C. After cryoprotection with 30% sucrose-PBS, tissue samples were embedded in OCT compound (Tissue Tek). Samples were sectioned at 14 µm and then mounted as above.

### Non-radioactive *in situ* hybridization

ISH on PFA-fixed cryosections was performed according to standard protocols for mouse [75], chicken, zebrafish [76] and axolotl [77]. Briefly, serial sections were hybridized with appropriate species-specific digoxygenin (DIG) labelled cRNA probes (see below) at a concentration of 0.5 ng/µl for 16 h at 70°C. Stringency washes were performed at 70°C. The sections were then incubated with anti-DIG antibody (1∶4,000; Roche Molecular Biochemicals, Mannheim, Germany) for 16 h at 4°C. After several washing steps the reaction was visualized using NBT-BCIP substrate (Roche Molecular Biochemicals) giving a blue reaction product. After stopping the reaction by several washes in PBS. Sections were counterstained with 4,6-diamidino-2-phenylindole (DAPI; 1 µg/ml; Molecular Probes). Sections were then rinsed with PBS followed by a quick rinse in dH_2_O. Then the slides were either mounted with Kaiser's Glycerol-Gelatin (Merck, Darmstadt, Germany) or were further processed for immunohistrochemistry (IHC; see below). From every specimen at a given time of development, we routinely processed more than four sections per marker. For each developmental stage, more than three animals were sacrificed.

### Probes for *in situ* hybridization

Antisense complementary ribonucleic acid (cRNA) probes were generated using DIG labelling mix (Roche Molecular Biochemicals). To synthesize murine prominin-1 cRNA probe a 2.1 kb cDNA fragment (nt 198–2,264; AF026269) was used. To synthesize cRNA probes from non-mammalian vertebrates the entire cDNA insert was used containing the full coding sequence of *Dr* prominin-1a (HQ386793), *Am* prominin-1 (DQ285041) and *Gg* prominin-1 (HQ386789), except for prominin-1b (nt 234–1,599; AF373869). The average homology of the *Dr* prominin-1 co-paralogues along the stretch used for ISH is ≈62%. Combined with the stringent hybridization and post-hybridization conditions, this precludes any cross-hybridization. To generate templates for murine *Crx* [35] and *Dr musashi-1* probes, cDNA clones IMAGE 4527863 (BC016502) and 7074855 (BC090916) were obtained from the German Resource Centre and Primary Database (German Science Centre for Genome Research, RZPD, Berlin). For in vitro transcription the entire insert was used containing the full coding sequence of the given molecule indicated above.

### Combined *in situ* hybridization and immunohistochemistry

Combined ISH/IHC was performed as previously described [78]. Briefly, after completion of the ISH, the slides of axolotl and zebrafish eyes were incubated with a mouse monoclonal anti-PCNA antibody (1∶500; Clone PC10, M0879; DAKO Cytomation; Glostrup, Denmark) followed by biotinylated-conjugated horse anti-mouse antibody (1∶500; Vector Laboratories, Burlingame, CA, USA). The latter was detected using Avidin-biotin-peroxidase complex (ABC Elite Vectastain kit; Vector Laboratories) and 3,3′-diaminobenzidine (DAB; 2 µg/ml: Fluka, Darmstadt, Germany) chromogen according to the manufacturer's instruction.

In order to detect Pax-6 in zebrafish retina, a purified rabbit antiserum against Pax-6 (1∶200; PRB-278P; Covance Inc.; Emeryville, CA) was used. The primary antibody was detected using an Alexa-546-conjugated goat anti-rabbit antibody (1∶750; Molecular Probes). The slides were mounted with Mowiol 4.88 (Calbiochem).

### Light and epifluorescence microscopies

Stained serial sections were observed using an Olympus BX61 microscope with the IPLAB software. The composite images were prepared from the digital data files using Adobe® Photoshop and Illustrator software (San Jose, CA).

### Cell culture and transfection

CHO cells were cultured as described previously [63] and transiently transfected at 50% confluence with pEGFP-N1-zfprom-1a-HA and pEGFP-N1-zfprom-1b-HA plasmids using nucleofection method with solution T and program #U-23 (Amaxa, Cologne, Germany). To enhance the expression of the transgene, cells were incubated with 5 mM sodium butyrate for 17 h. Under these conditions, 30–50% cells expressed recombinant prominin-1.

### Membrane preparation

Transfected CHO cells were scraped with a rubber policeman in ice-cold PBS and collected by centrifugation at 1.200× g for 5 min at 4°C. Cell pellets were homogenized with a glass-Teflon pestle in sucrose buffer (300 mM sucrose, 5 mM EDTA, 10 mM Hepes-KOH, pH 7,5 and Complete Protease Inhibitor (CPI; Roche Molecular Biochemicals)) at 4°C, and subsequently incubated for 30 min on ice. Lysates were centrifuged at 1.000× g for 10 min at 4°C, and the resulting supernatants containing membranes were subjected to ultracentrifugation at 60.000× g for 60 min at 4°C. The pellets were resuspended in 1% SDS/20 mM sodium phosphate and boiled for 2 min at 95°C. Samples were either subjected to endoglycosidase treatment (see below), or after adding Laemmli buffer (4X) they analyzed by SDS-polyacrylamide-gel electrophoresis (SDS-PAGE) and immunoblotting.

### Zebrafish retina lysate

Eyes from adult zebrafish were collected in PBS on ice. After a circumferential incision at the corneo-scleral border, the anterior segment of the eye with the lens and the vitreous body were removed. The posterior eye-cup was further dissected with fine-tip watchmakers forceps and the retina was freed from *choroidea* and *sclera*. Isolated retinas were washed with sterile ice-cold PBS containing CPI (Roche Molecular Biochemicals). The tissue was subsequently homogenized with a Teflon pestle in RIPA lysis buffer (1% NP-40, 0.5% sodium-deoxycholate, 0.1% SDS, 0.15 M NaCl, 0.05 M Tris-HCl, pH 8) containing CPI. Lysates were further incubated on ice for 15 min prior centrifugation at 1.000× g for 10 min at 4°C. Supernatants were boiled at 95°C as described above, and samples processed for endoglycosidase digestion and immunoblotting.

### Endoglycosidase digestion and immunoblotting

Membrane extracts of CHO cells (corresponding to one-fifth of a T75 flask) or tissue lysates prepared from zebrafish retina were incubated in the absence or presence of 1 U peptide-N-glycosidase F (PNGase F) overnight at 37°C according to the manufacturer's instructions (Roche Molecular Biochemicals). Protein samples were subjected to SDS-PAGE (7.5%) and transferred polyvinylidene difluoride (PVDF) membranes (pore size: 0.45 µm; Millipore Corp.; Belford, MA) using a semi-dry transfer cell system (Cti; Idstein, Germany). After transfer, immunoblotting was performed as previously described [64]. Zebrafish prominin-1a and prominin-1b and the recombinant HA-tagged proteins were detected using either rabbit antiserum 6F44 (1∶1,000; prominin-1a) or antiserum 43C9 (1∶1,000; prominin-1b) or rat monoclonal anti-HA antibody (1∶1,1000; Roche Molecular Biochemicals), respectively. In all cases, antigen-antibody complexes were detected using appropriate horseradish peroxidase-conjugated secondary antibodies (Dianova: Hamburg, Germany) and these were visualized with enhanced chemiluminescence developing reagents (ECL System: Amersham Biosciences; Poscataway, NJ).

## Supporting Information

Figure S1 **Localization of prominin-1 in the eye of chick at the embryonic day 15.** (A–E′) Cryosections of chick embryos (E15) were processed for non-radioactive ISH using either antisense (A–C, E; *Prom1*) or sense (D; *Prom-1S*) DIG-labeled prominin-1 probe. Sections were counterstained with DAPI (A′, B′, E′). The boxed areas in B are shown at higher magnification in panel C. (A) White arrowheads indicate a weak expression of prominin-1 at the inner layer of the anterior part (*pars plicata*) of the prospective ciliary epithelium. (B, C) Dashed lines indicate the putative border between prospective blind part (*pars plana, pp*), the ciliary marginal zone (CMZ) and peripheral sensory part of the retina. White arrowheads indicate the prominin-1 expression in all three regions. Black asterisk indicates *prominin-1* transcripts in mesenchymal cells giving rise to the ciliary body. (E) White and black arrowheads indicate the prominin-1 expression in the outer nuclear layer (ONL) and scleral side of the inner nuclear layer (INL), respectively, in the prospective sensory retina. Hollow arrowhead indicates the lack of prominin-1 signal in the vitreal side of INL. Asterisk, retinal pigmented (dark brown) epithelium; GCL, ganglion cell layer. Scale bars, A, C, E; 25 µm; B, D; 50 µm.
(TIF)

Figure S2 **Localization of prominin-1 in the eye of chicken at the embryonic day 20.** (A–E′) Cryosections of chicken embryos (E20) were processed for non-radioactive ISH using either antisense (A, B, D, E; *Prom1*) or sense (C; *Prom-1S*) DIG-labeled prominin-1 probe. Sections were counterstained with DAPI (A′, D′, E′). The boxed areas in A are shown at higher magnification in panel B. (A, B) Dashed lines indicate the putative border between prospective blind part (*pars plana, pp*), the ciliary marginal zone (CMZ) and sensory part of the retina. White arrowheads indicate the expression of prominin-1 in all three regions. (D, E) White and black arrowheads indicate the prominin-1 expression in the outer nuclear layer (ONL) and scleral side of the inner nuclear layer (INL), respectively, in regions located at the periphery (D) and center (E) of prospective sensory retina. Hollow arrowhead indicates the lack of prominin-1 signal in the vitreal side of INL. Asterisk, retinal pigmented (dark brown) epithelium; GCL, ganglion cell layer. Scale bars, A, 50 µm; B–E, 25 µm.
(TIF)

Table S1 Alternative splice variants of *A. mexicanum* prominin-1.
(DOC)

Table S2 Alternative splice variants of *G. gallus* prominin-1.
(DOC)

Table S3 Alternative splice variants of *Danio rerio* prominin-1a.
(DOC)

Table S4 Pairwise comparison of prominin-1.s11 sequences between species.
(DOC)

Table S5 Summary of prominin–1 splice variants and their alternative exons.
(DOC)

Materials and Methods S1 Cloning of prominin-1 from non-mammalian vertebrates. Computer analysis.
(DOC)
