# Supplementary figures and images for: Distinct and Conserved Prominin-1/CD133–Positive Retinal Cell Populations Identified across Species
Source: PLoS One. 2011 Mar 2;6(3):e17590. doi: 10.1371/journal.pone.0017590 (PMC3047580; doi:10.1371/journal.pone.0017590)

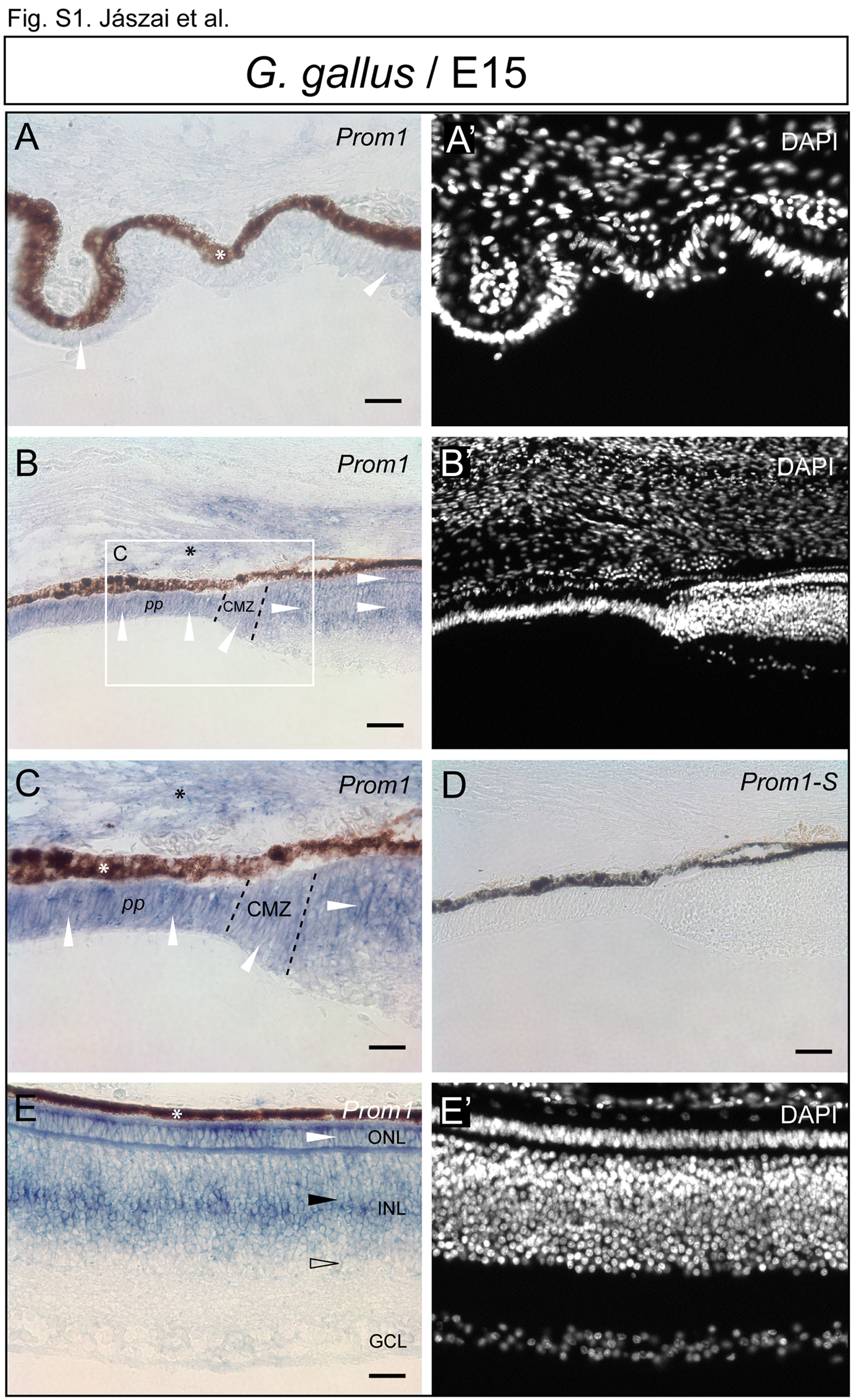

Supplement: Figure S1 — Localization of prominin-1 in the eye of chick at the embryonic day 15. (A–E′) Cryosections of chick embryos (E15) were processed for non-radioactive ISH using either antisense (A–C, E; Prom1) or sense (D; Prom-1S) DIG-labeled prominin-1 probe. Sections were counterstained with DAPI (A′, B′, E′). The boxed areas in B are shown at higher magnification in panel C. (A) White arrowheads indicate a weak expression of prominin-1 at the inner layer of the anterior part (pars plicata) of the prospective ciliary epithelium. (B, C) Dashed lines indicate the putative border between prospective blind part (pars plana, pp), the ciliary marginal zone (CMZ) and peripheral sensory part of the retina. White arrowheads indicate the prominin-1 expression in all three regions. Black asterisk indicates prominin-1 transcripts in mesenchymal cells giving rise to the ciliary body. (E) White and black arrowheads indicate the prominin-1 expression in the outer nuclear layer (ONL) and scleral side of the inner nuclear layer (INL), respectively, in the prospective sensory retina. Hollow arrowhead indicates the lack of prominin-1 signal in the vitreal side of INL. Asterisk, retinal pigmented (dark brown) epithelium; GCL, ganglion cell layer. Scale bars, A, C, E; 25 µm; B, D; 50 µm. (TIF) [file pone.0017590.s001.tif]

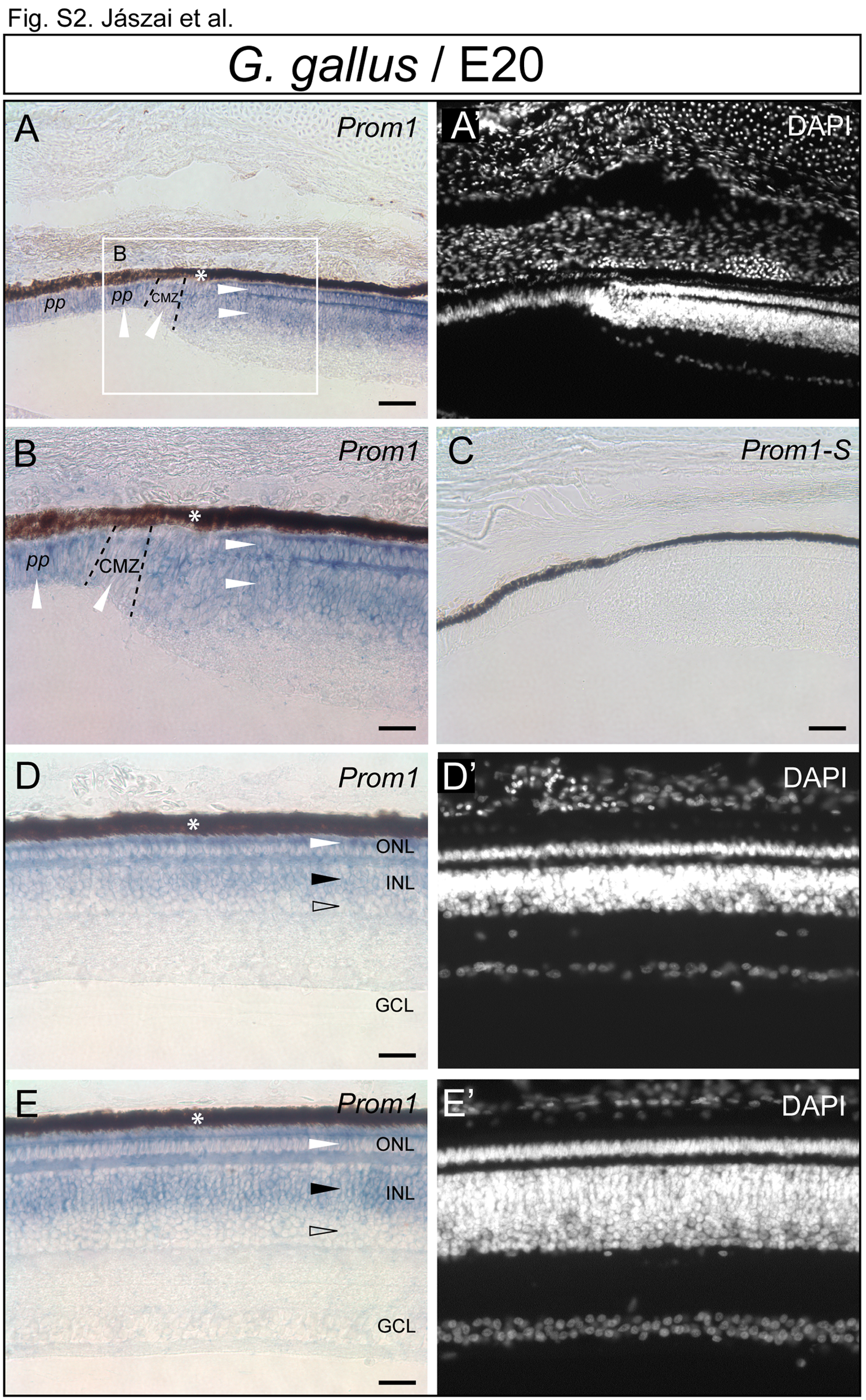

Supplement: Figure S2 — Localization of prominin-1 in the eye of chicken at the embryonic day 20. (A–E′) Cryosections of chicken embryos (E20) were processed for non-radioactive ISH using either antisense (A, B, D, E; Prom1) or sense (C; Prom-1S) DIG-labeled prominin-1 probe. Sections were counterstained with DAPI (A′, D′, E′). The boxed areas in A are shown at higher magnification in panel B. (A, B) Dashed lines indicate the putative border between prospective blind part (pars plana, pp), the ciliary marginal zone (CMZ) and sensory part of the retina. White arrowheads indicate the expression of prominin-1 in all three regions. (D, E) White and black arrowheads indicate the prominin-1 expression in the outer nuclear layer (ONL) and scleral side of the inner nuclear layer (INL), respectively, in regions located at the periphery (D) and center (E) of prospective sensory retina. Hollow arrowhead indicates the lack of prominin-1 signal in the vitreal side of INL. Asterisk, retinal pigmented (dark brown) epithelium; GCL, ganglion cell layer. Scale bars, A, 50 µm; B–E, 25 µm. (TIF) [file pone.0017590.s002.tif]
