# Supplementary material for: Distinct and Conserved Prominin-1/CD133–Positive Retinal Cell Populations Identified across Species
Source: PLoS One. 2011 Mar 2;6(3):e17590. doi: 10.1371/journal.pone.0017590 (PMC3047580; doi:10.1371/journal.pone.0017590)
Supplement: Table S1 — Alternative splice variants of A. mexicanum prominin-1. (DOC) [file pone.0017590.s003.doc]

**Table S1. Alternative splice variants of *A. mexicanum* prominin-1**

| GenBank  (accession number) | Inclusion of facultative exons^§^ | | | | | | | Splice variant designation^§^ |
| --- | --- | --- | --- | --- | --- | --- | --- | --- |
|  | 3 | 9 | **A10’**^*^ | 19 | 26b | 27 | 28 |  |
| DQ285041 | – | + | – | – | + | + | + | s1 |
| DQ285045 | + | + | – | – | – | – | + | s11 |
| DQ285042 | – | + | – | + | + | + | + | **s13** |
| DQ285043 | + | + | – | + | + | – | + | **s14** |
| DQ285044 | + | + | + | + | – | – | + | **s15** |

Presence (+) or absence (–) of a facultative exon in a given prominin-1 splice variant.

§See Fargeas et al., (2007), newly described exon and splice variants appear in bold.

*ALPY insert located between exon 10 and 11 according to the mammalian gene structure. The letter A refers to amphibian, and number 10’ to its relative position with respect to the exon numbering adopted for primate and rodent prominin-1 genes (Fargeas et al. 2007).
