# Supplementary material for: Distinct and Conserved Prominin-1/CD133–Positive Retinal Cell Populations Identified across Species
Source: PLoS One. 2011 Mar 2;6(3):e17590. doi: 10.1371/journal.pone.0017590 (PMC3047580; doi:10.1371/journal.pone.0017590)
Supplement: Table S2 — Alternative splice variants of G. gallus prominin-1. (DOC) [file pone.0017590.s004.doc]

**Table S2. Alternative splice variants of *G. gallus* prominin-1**

| GenBank  (accession number) | Inclusion of facultative exons^§^ | | | | | | | | | Splice variant designation^§^ |
| --- | --- | --- | --- | --- | --- | --- | --- | --- | --- | --- |
|  | 3 | 9 | **A10’**^‡^ | 19 | 25 | 26a | 26b | 27 | 28 |  |
| HQ286791 | – | + | – | – | – | – | – | – | – | s7 |
| HQ286789 | + | + | – | – | – | – | – | – | + | s11 |
| HQ286792 | – | + | – | – | – | – | – | + | + | **s16** |
| HQ286790 | + | + | + | – | – | – | – | + | + | **s17** |
| Predicted (XM_001232164) | + | + | + | – | – | – | + | + | + |  |
| EST (BU448576) | nd | nd | nd | – | – | – | + | + | + |  |

Presence (+) or absence (–) of a facultative exon in a given prominin-1 splice variant.

§See Fargeas et al., (2007), newly described exon and splice variants appear in bold.

‡See Table S1.

nd; not determined.
