# Supplementary material for: Distinct and Conserved Prominin-1/CD133–Positive Retinal Cell Populations Identified across Species
Source: PLoS One. 2011 Mar 2;6(3):e17590. doi: 10.1371/journal.pone.0017590 (PMC3047580; doi:10.1371/journal.pone.0017590)
Supplement: Table S3 — Alternative splice variants of Danio rerio prominin-1a. (DOC) [file pone.0017590.s005.doc]

**Table S3. Alternative splice variants of *Danio rerio* prominin-1a**

| GenBank  (accession number) | Inclusion of facultative exons^§^ | | | | | | | | | | | Splice variant designation^§^ |
| --- | --- | --- | --- | --- | --- | --- | --- | --- | --- | --- | --- | --- |
|  | 3 | **F7’^*^** | 9 | **A10’**^‡^ | 19 | 25 | 26a | 26b | 27 | **F27’^*^** | 28 |  |
| HQ386793 | + | – | + | – | – | – | – | – | – | – | + | s11 |
| HQ386794 | + | + | + | – | – | – | – | – | – | – | + | **s18** |
| HQ386796 | – | – | + | – | + | – | – | + | – | + | {+} | **s19** |
| HQ386795 | – | – | + | – | – | – | – | + | – | + | {+} | **s20** |
| clone MGC:194953 IMAGE:9038469  (NM_001115143) | – | – | + | – | – | – | – | – | – | – | – | s6 |

Presence (+) or absence (–) of a facultative exon in a given prominin-1 splice variant.

§See Fargeas et al., (2007), newly described exons and splice variants appear in bold.

*New exons F7’ (IKVEKAIK) and F27’ (IPTYDTMTRFPRASAPPRHADW). The letter F refers to fish, and numbers 7’ and 27’ to their relative position with respect to the exon numbering adopted for primate and rodent prominin-1 genes (Fargeas et al. 2007). F27’ harbors an in-frame stop codon, the downstream exon 28 (braced) is therefore not translated in s19 and s20.

‡ See Table S1.
