# Supplementary material for: Distinct and Conserved Prominin-1/CD133–Positive Retinal Cell Populations Identified across Species
Source: PLoS One. 2011 Mar 2;6(3):e17590. doi: 10.1371/journal.pone.0017590 (PMC3047580; doi:10.1371/journal.pone.0017590)
Supplement: Table S4 — Pairwise comparison of prominin-1.s11 sequences between species. (DOC) [file pone.0017590.s006.doc]

Table S4. Pairwise comparison of prominin-1.s11 sequences between species

| *Identity / Similarity  (%) | Homo sapiens | Mus musculus | Gallus gallus | Ambystoma mexicanum | Danio rerio ^§^(1a) |
| --- | --- | --- | --- | --- | --- |
| Danio rerio ^§^(1b) | 41 / 65 | 40 / 64 | 50 / 67 | 54 / 74 | 57 / 75 |
| Danio rerio ^§^(1a) | 43 / 66 | 40 / 65 | 59 / 78 | 55 / 75 |  |
| Ambystoma mexicanum | 48 / 70 | 45 / 67 | 70 / 86 |  | |
| Gallus gallus | 52 / 73 | 47 / 50 |  | | |
| Mus musculus | 60 / 78 |  | | | |

*The analysis was performed using Align program via global alignment with BLOSUM 62 matrix, gap open and gap extend penalties at 10 and 0.5, respectively.

^§^*Dr* prominin-1a or prominin-1b. Note that 1b is actually splice variant s21.
