# Supplementary material for: Distinct and Conserved Prominin-1/CD133–Positive Retinal Cell Populations Identified across Species
Source: PLoS One. 2011 Mar 2;6(3):e17590. doi: 10.1371/journal.pone.0017590 (PMC3047580; doi:10.1371/journal.pone.0017590)
Supplement: Table S5 — Summary of prominin–1 splice variants and their alternative exons. (DOC) [file pone.0017590.s007.doc]

Table S5. Summary of prominin–1 splice variants and their alternative exons

| Splice variant | ^¶^C–terminus type | Inclusion of facultative exons | | | | | | | | | | |
| --- | --- | --- | --- | --- | --- | --- | --- | --- | --- | --- | --- | --- |
|  |  | 3 | F7’ | 9 | A10’ | 19 | 25 | 26a | 26b | 27 | F27’ | 28 |
| s1 | A | – | – | + | – | – | – | – | + | + | – | + |
| s2 | A | + | – | + | – | – | – | – | + | + | – | + |
| s3 | B | – | – | + | – | – | – | + | (+) | (+) | – | (+) |
| s4 | B | – | – | – | – | – | – | + | (+) | (+) | – | (+) |
| s5 | B | – | – | – | – | – | – | + | (+) | (+) | – | (+) |
| s6 | C | – | – | + | – | – | – | (+) | (+) | (+) | – | (+) |
| s7 | D | – | – | + | – | – | – | – | – | – | – | + |
| s8 | D | + | – | + | – | + | – | – | – | – | – | + |
| s9 | E | – | – | + | – | – | + | – | + | ND | ND | ND |
| s10 | F | – | – | + | – | – | – | – | + | – | – | + |
| s11 | D | + | – | + | – | – | – | – | – | – | – | + |
| s12 | F | + | – | + | – | – | – | – | + | – | – | + |
| s13 | A | – | – | + | – | + | – | – | + | + | – | + |
| s14 | F | + | – | + | – | + | – | – | + | – | – | + |
| s15 | D | + | – | + | + | + | – | – | – | – | – | + |
| s16 | G | – | – | + | – | – | – | – | – | + | – | + |
| s17 | G | + | – | + | + | – | – | – | – | + | – | + |
| s18 | D | + | + | + | – | – | – | – | – | – | – | + |
| s19 | H | – | – | + | – | + | – | – | + | – | + | (+) |
| s20 | H | – | – | + | – | – | – | – | + | – | + | (+) |
| ^§^s21 | D | + | – | + | + | – | – | – | – | – | – | + |

^§^*Dr* prominin-1b.

^¶^ For the sequence information see Table 1.

Brackets indicated that the exon is not translated.

ND, not determined.
