# Supplementary material for: Distinct and Conserved Prominin-1/CD133–Positive Retinal Cell Populations Identified across Species
Source: PLoS One. 2011 Mar 2;6(3):e17590. doi: 10.1371/journal.pone.0017590 (PMC3047580; doi:10.1371/journal.pone.0017590)
Supplement: Materials and Methods S1 — Cloning of prominin-1 from non-mammalian vertebrates. Computer analysis. (DOC) [file pone.0017590.s008.doc]

**Supplementary Information**

**Supplemental Materials and Methods**

Cloning of prominin-1 from non-mammalian vertebrates

*Axolotl*

In the absence of expressed sequence tags (EST) encoding for an *Am* prominin-1 from public databases or *A. tigrinum* sequencing project (University of Kentucky; Lexington) a degenerated PCR cloning strategy was applied. Two homologous stretches deduced from the multiple sequence alignment of both cloned and predicted vertebrate prominin-1 enabled the design of degenerated oligonucleotides 5’-CTGTRACAAMTGTGGTGGVGARATGC-3’ [F-jj-520] and 5’-TCAACATCATCATANACATCCTCHGWRTCC-3’ [R-jj-524] as 5’ and 3’ primers, respectively. The wobbling nucleotide positions (letter underlined) are indicated according to the convention. Total RNA prepared from whole 3.5–cm juvenile axolotl was used to prepare first strand cDNA template with Superscript II reverse transcriptase (Gibco-BRL/Invitrogen) for degenerate PCR reaction. As a result, a 2.1-kb fragment of *Am* prominin-l was amplified. However, it did not contain the 5’- and the 3’-ends. The missing extremities were amplified by 5’- and 3’-RACEs using SMART RACE cDNA synthesis kit (#634914, Clontech) according to the manufacturer’s instructions. The gene-specific oligonucleotides used for the first and second round of amplification of 5’-RACE were 5’-CAGCTTGCGCTTGCTTGCATGCTGGATCAC-3’ [GSP, jj-578] and 5’-GTCGTTCGGTTCCTTCCTTCGTCGCCTCCGCAG-3’ [NGSP, jj-579]. The gene-specific oligonucleotides used for the first and second round of amplification of 3’-RACE were 5’-GTCACGCAAACAGCATTCGGATGATCCATCTG-3’ [GSP, jj-583] and 5’-GAGTGTTCGAGTACTTCAGAGATCATCAGTG-3’ [NGSP, jj-582]. Reaction products were gel extracted (Qiagen GmBH, Helden, Germany) and subcloned into pCRII-TOPO TA vector (Invitrogen). The identity of the inserts was determined by sequencing. Finally, the entire *Am* prominin-1 coding sequence was amplified with Platinum Pfx proof-reading DNA polymerase (Gibco-BRL) using the following oligonucleotides 5’-CTCTAGCCATGGCCGCAGGGCTC-3’ [F-jj-610] and 5’-GAGGTCTTTTCCGTATCCTCTACCACTG-3’ [R-jj-615] as 5’ and 3’ primers, respectively. The same cDNA pool described above was used as template. The Under these conditions, we have identified five distinct splice variants of *Am* prominin-1 named s1, s11, s13, s14 and s15 according to the proposed nomenclature of prominin molecules [1]. Their sequences were deposited in GenBank database under the following accession numbers DQ285041, DQ285045, DQ284042, DQ284043 and DQ284044, respectively.

*Chick*

With the sequence information gained from the multiple alignment of prominin molecules, we amplified a prominin-1 cDNA fragment from both E2 and E3 (HH14 and HH20) chick cDNA templates using the following degenerated oligonucleotides 5’-ACHACMMGVGGCTGYNTBTCCACCAC-3’ [F-jj-521] and 5’-TCAACATCATCATANACATCCTCHGWRTCC-3’ [R-jj-524] as 5’- and 3’ primer, respectively. The wobbling nucleotide positions (letter underlined) are indicated according to the convention. Reaction products were purified and subcloned into pCRII-TOPO TA vector (Invitrogen). The cDNA insert (~1.1 Kb) was sequenced and its information was used to identify a genomic contig containing *Gg* prominin-1. Its pair-wise sequence alignment with either murine or *Am* prominin-1 allowed the prediction of the putative 5’- and 3’-ends of *Gg* prominin-1. Based on them, the entire coding sequence was amplified from E5 (HH27) chick eye or brain cDNA templates using the following oligonucleotides 5’-GTTAGCCATGGCTGTGGAACTCTG-3’ [F-jj-734] and 5’-CAGTTTCTGTTACCACTGTTCCAC-3’ [R-jj-736] as 5’ and 3’ primers, respectively. PCR products were subcloned into pCR4Blunt-TOPO (K287520; Invitrogen) and sequenced. Under these conditions, in addition to s7 and s11 prominin-1 splice variants two novel ones were identified and named s16 and s17. Two of them (s11, s17) were derived from the eye while the others (s7, s16) from brain cDNA templates. Their sequences were deposited in GenBank database under accession numbers HQ386789, HQ386791, HQ386792, and HQ386790, respectively.

*Zebrafish*

We have previously identified two EST sequences (AF160970, AF373869) encoding for distinct zebrafish homologues of the mammalian prominin-1 [2]. They were initially referred to as *Dr* prominin-like 1 and 2, and renamed since 2008 following the zebrafish nomenclature guidelines ([http://zfin.org](http://zfin.org/)) prominin-1a and b, respectively. Their clones are derived from adult liver and eye, respectively. Although, prominin-1b contained an entire coding sequence, prominin-1a was an incomplete clone missing approximately a third of the 3’-end of the coding sequence [2]. Its missing 3’-end was obtained by 3’-RACE using a cDNA pool prepared from adult retina with SMART RACE cDNA synthesis kit. The gene-specific oligonucleotides used for the first and second round of amplification were 5’-TGGTCAACTCTGCTTGGAGAAACTTCATACCAG-3’ [GSP; jj-586] and 5’-GATACCTGTACAATGACCCAGAGATGGACCTTACAG-3’ [NGSP; jj-587a]. Finally, the entire *Dr* prominin-1a was amplified from either adult retina or brain cDNA templates using the following oligonucleotides 5’- AGCGGAGCATCAGACTGACTGAC-3’ [F-jj-642] and 5’- GTCAGAGCTCGTTGAGGGACAG-3’ [R-jj-646] as 5’ and 3’ primers, respectively. PCR products were gel extracted and subcloned into pCR4-TOPO blunt cloning vector (Invitrogen). The identity of the inserts was confirmed by sequencing. Under these conditions, we have identified four distinct *Dr* prominin-1a splice variants named s11, s18, s19 and s20. Two of them (s11, s18) were derived from the retina while the others (s19, s20) from brain cDNA pools. Their sequences were deposited in GenBank database under the following accession numbers HQ386793 HQ386794, HQ386796 and HQ386795, respectively.

All sequencing reactions were performed using Applied Biosystems 3730 Genetic Analyzer at the MPI-CBG facility (Dresden, Germany).

Computer analysis

The nucleotide and protein sequences were analyzed by the HUSAR compilation of sequence analysis tools accessible on-line at the German Cancer Research Center (Deutsches Krebsforschungszentrum, Heidelberg, Germany; http://genome.dkfz-heidelberg.de/) or European molecular Biology Laboratory–European Bioinformatics Institutes (EMBL-EBI; http://www.ebi.ac.uk/). Pairwise protein sequence comparisons were performed using the ALIGN program with a BLOSUM 62 matrix [3] and multiple sequence alignments using the PileUP multiple sequence alignment.

**Supplemental References**

1. Fargeas CA, Huttner WB, Corbeil D (2007) Nomenclature of prominin-1 (CD133) splice variants – an update. Tiss Antig 69: 602-606.

2. Fargeas CA, Florek M, Huttner WB, Corbeil D (2003) Characterization of prominin-2, a new member of the prominin family of pentaspan membrane glycoproteins. J Biol Chem 278: 8586-8596.

3. Myers EW, Miller W (1988) Optimal alignments in linear space. Comp Appl Biosci 4(1): 11-17.
